# Exercise Limitation in Children and Adolescents With Severe Refractory Asthma: A Lack of Asthma Control?

**DOI:** 10.3389/fphys.2020.620736

**Published:** 2021-01-26

**Authors:** Rita C. Faleiro, Eliane V. Mancuzo, Fernanda C. Lanza, Mônica V. N. P. Queiroz, Luciano F. L. de Oliveira, Vinicius O. Ganem, Laura B. Lasmar

**Affiliations:** ^1^Faculdade de Medicina, Universidade Federal de Minas Gerais, Belo Horizonte, Brazil; ^2^School of Physical Education, Physiotherapy and Occupational Therapy, Federal University of Minas Gerais, Belo Horizonte, Brazil

**Keywords:** exercise-induced asthma, exercise test, cardiopulmonary exercise test, aerobic capacity, severe asthma

## Abstract

**Background:**

Patients with severe refractory asthma (SRA), even when using high doses of multiple controller medications in a regular and appropriate way, can have persistent complaints of exercise limitation.

**Methods:**

This was a cross-sectional study involving patients with SRA (treated with ≥ 800 μg of budesonide or equivalent, with ≥ 80% adherence, appropriate inhaler technique, and comorbidities treated), who presented no signs of a lack of asthma control other than exercise limitation. We also evaluated healthy controls, matched to the patients for sex, age, and body mass index. All participants underwent cardiopulmonary exercise testing (CPET) on a cycle ergometer, maximum exertion being defined as ≥ 85% of the predicted heart rate, with a respiratory exchange ratio ≥ 1.0 for children and ≥ 1.1 for adolescents. Physical deconditioning was defined as oxygen uptake (VO_2_) < 80% of predicted at peak exercise, without cardiac impairment or ventilatory limitation. Exercise-induced bronchoconstriction (EIB) was defined as a forced expiratory volume in one second ≥ 10% lower than the baseline value at 5, 10, 20, and 30 minutes after CPET.

**Results:**

We evaluated 20 patients with SRA and 19 controls. In the sample as a whole, the mean age was 12.9 ± 0.4 years. The CPET was considered maximal in all participants. In terms of the peak VO_2_ (VO_2__peak_), there was no significant difference between the patients and controls, (*P* = 0.10). Among the patients, we observed isolated EIB in 30%, isolated physical deconditioning in 25%, physical deconditioning accompanied by EIB in 25%, and exercise-induced symptoms not supported by the CPET data in 15%.

**Conclusion and Clinical Relevance:**

Physical deconditioning, alone or accompanied by EIB, was the determining factor in reducing exercise tolerance in patients with SRA and was not therefore found to be associated with a lack of asthma control.

## Introduction

Children and adolescents with asthma should be encouraged to engage in regular physical activity; one of the objectives of adjusting the treatment regimen is to allow the patients to engage in such activities without any limitations (Global Initiative for Asthma, 2020). Before stepping up the pharmacological treatment in patients with asthma, it is advisable to perform a general clinical reassessment of adherence to treatment, inhaler technique, the environment, and comorbidities. However, even after all of those basic factors have been optimized, some patients continue to complain of exercise-induced symptoms, which require investigation (McNicholl et al., 2011; Global Initiative for Asthma, 2020).

Cardiopulmonary exercise testing (CPET) is the gold standard for the investigation of exercise limitation, which, in individuals with asthma, can be attributed to airway obstruction, ventilatory limitation, an increased level of perception of dyspnea, or exercise-induced bronchoconstriction (EIB) (Weisman et al., 2003; Minic and Sovtic, 2017). For the diagnosis of EIB, CPET is performed in conjunction with spirometry, and the best way to estimate exercise capacity is by measuring the oxygen uptake (VO_2_) (Weisman et al., 2003; Parsons et al., 2013). A reduction in VO_2_, at the end of the test, indicates a reduction in aerobic capacity, which could be due to ventilatory limitation, with or without EIB, cardiac impairment, or physical deconditioning (Weisman et al., 2003).

Studies attempting to determine whether children and adolescents with asthma are less active than are their healthy peers have produced conflicting results. In some studies, asthma was not associated with physical inactivity (Anthracopoulos et al., 2012; Cassim et al., 2016; Pike et al., 2019), whereas other studies have shown that, among individuals with asthma, a reduction of physical activity occurs only in those who have EIB (van der Kamp et al., 2019), have recently been hospitalized (Pike et al., 2019), have asthma that is more severe or in uncontroled (Vahlkvist and Pedersen, 2009; Sousa et al., 2014; Matsunaga et al., 2017), or are obese (Anthracopoulos et al., 2012; Sousa et al., 2014). In addition to these factors, the time spent in sedentary activities and less physical conditioning, as determined by quantifying the cumulative duration of continuous exercise, has been associated with wheezing during exercise in young children (Lu et al., 2020). The symptoms that limit physical activity are non-specific and may be secondary to conditions other than asthma, such as vocal cord dysfunction, dysfunctional breathing, and physical deconditioning, which makes the investigation more complex (Seear et al., 2005; Pedersen and Mozun, 2020).

Although some studies have evaluated the level of physical activity in patients with asthma using objective measures (Cassim et al., 2016; Pike et al., 2019; van der Kamp et al., 2019) few have evaluated physical deconditioning (Vahlkvist and Pedersen, 2009; Villa et al., 2011; Schiwe et al., 2019) or have used CPET to investigate the mechanisms responsible for reducing exercise tolerance (McNicholl et al., 2011). To our knowledge, there have been no studies employing CPET to analyze the possible mechanisms involved in exercise limitation in pediatric patients with severe asthma.

The present study aims to determine the causes of exercise limitation in children and adolescents with severe asthma that was refractory to regular, appropriate pharmacological treatment. The specific focus was on exercise limitation as the only manifestation of a lack of asthma control.

## Materials and Methods

### Patients

This was a cross-sectional study, nested within a prospective cohort study of patients with severe asthma, carried out between May 2018 and March 2020 at the Multidisciplinary Center for Patients with Difficult-to-Control Asthma of the *Hospital das Clínicas* and the Laboratory for Evaluation and Research in Cardiorespiratory Performance, both of the Federal University of Minas Gerais, in the city of Belo Horizonte, Brazil. All medications necessary for the treatment of the children and adolescents with asthma, as well as the spacers for the inhalers, are made available free of charge via the “Wheezy Child” Program of the city of Belo Horizonte and via the Minas Gerais State Public Health Care System (Lasmar et al., 2009). The patients were receiving treatment with dry-powder inhalers delivering the budesonide-formoterol combination—Symbicort (AstraZeneca, Lund, Sweden) or Alenia (Aché Laboratórios Farmacêuticos SA, Guarulhos, Brazil)—or with dry-powder or metered-dose inhalers containing one of the following: fluticasone with salmeterol (Seretide; GlaxoSmithKline, Stevenage, England); montelukast (Montelair; Aché Laboratórios Farmacêuticos SA); tiotropium bromide (Spiriva Respimat; Boehringer Ingelheim, Brazil); or omalizumab (Xolair; Novartis Biocências SA, São Paulo, Brazil).

In accordance with the protocol of the facility (Versiani et al., 2017), which was adapted from the Global Initiative for Asthma (GINA) criteria (Pendersen et al., 2017), when a patient is admitted, all necessary tests are performed to exclude alternative diagnoses, as is spirometry with a bronchodilator test to confirm the diagnosis of asthma (Pellegrino et al., 2005). Patients are reevaluated every 3–4 months, the dose of the medication(s) being adjusted, according to the level of control, after reevaluation of the inhaler technique, environmental control, and comorbidities.

From among the patients in the cohort, we selected 24 with severe refractory asthma. We included only those who consistently complained of exercise limitation, despite receiving optimized therapy (step 4 or 5 treatment), exhibiting appropriate inhaler techniques, having no unaddressed comorbidities, having a confirmed rate of treatment adherence ≥ 80% in the last 4 months, and having had no other complaints indicative of a lack of asthma control. We determined the rate of adherence to the use of the inhaled corticosteroid, in accordance with the protocol of the facility (Versiani et al., 2017), by calculating the proportion of doses used in relation to the expected number of doses, as determined from the dose counters of the devices or by counting the empty capsules (for the dry-powder inhalers), based on the recorded dates on which the medications were dispensed. Patients with symptoms of gastroesophageal reflux disease were monitored by specialists and underwent diagnostic assessment if necessary. Patients suspected of having an emotional or behavioral disorder were referred to and monitored by specialists (Pendersen et al., 2017; Versiani et al., 2017). Vocal cord dysfunction was assessed on the basis of the clinical history, blunting of the inspiratory loop on spirometry before and after CPET, and symptoms during exercise (Pendersen et al., 2017). Participants who had exacerbations during the study procedures were excluded, as were those who had symptoms other than exercise limitation, those who were unable to understand the maneuvers required to perform spirometry or CPET, and those who had accompanying cardiovascular problems identified in clinical and diagnostic assessments (electrocardiogram and Doppler echocardiogram).

We also evaluated a group of 21 healthy controls, recruited from public and private schools in the city of Belo Horizonte, who were matched to the patients for sex, age, and body mass index. Asthma and allergic rhinitis were excluded by using the International Study of Asthma and Allergies in Childhood questionnaire (Beasley et al., 1998), on which a negative response to question 2 (“Has your child had wheezing or chest tightness in the last 12 months?”) and a total score < 6 were indicative of the absence of both conditions. None of the subjects in the control group were involved in exercise training programs other than recreational physical activities.

The study was approved by the Research Ethics Committee of the Federal University of Minas Gerais (Reference no. 3,720,392). All patients and their parents or legal guardians gave written informed consent.

### Procedures

In the patients, the level of asthma control was assessed by applying the GINA criteria, which involve questioning subjects about daytime and nighttime symptoms, use of short-acting bronchodilators and any activity limitation due to asthma (Pendersen et al., 2017). The results of that assessment allowed us to categorize the level of asthma control as follows: controlled (negative response to all four questions), partially controlled (negative response to one or two questions), or uncontrolled (positive response to three or four questions) (Pendersen et al., 2017). In addition, children over 12 years of age were assessed with the asthma control test (ACT) (Roxo et al., 2010), whereas those between 6 and 12 years of age were assessed with the childhood asthma control test (C-ACT) (Oliveira et al., 2016).

For the diagnosis of allergic rhinitis, we applied the criteria established in the Allergic Rhinitis and its Impact on Asthma guidelines (Bousquet et al., 2008). Skin prick tests were performed with various allergens (ALK-Abelló, Hørsholm, Denmark), as well as with positive and negative controls (histamine and saline, respectively). A wheal ≥ 3 mm larger than that for the negative control was considered indicative of positivity for allergic sensitization (Bousquet et al., 2012). The allergens tested were *Dermatophagoides pteronyssinus*, *Dermatophagoides farinae*, *Blomia tropicalis*, *Alternaria alternata*, *Aspergillus fumigatus*, cat epithelium, dog epithelium, and cockroach allergens (from *Periplaneta americana* and *Blattella germanica*).

### Spirometry

For the objective diagnosis of asthma, an increase in forced expiratory volume in one second (FEV_1_) of 12% or 200 mL after administration of a bronchodilator (400 μg of albuterol by metered-dose inhaler) in a previous spirometry examination was considered indicative (Pellegrino et al., 2005). For the identification of EIB, we used a Koko spirometer (Pulmonary Data Service, Inc., Louisville, CO, United States), performing spirometry, as recommended by the American Thoracic Society (Miller et al., 2005; Pellegrino et al., 2005), at 5, 10, 15, 20, and 30 minutes after CPET. A ≥ 10% reduction in FEV_1_ in one or more measurements during the 30-minute post-CPET period was considered indicative of EIB (Parsons et al., 2013). The severity of EIB can be graded as mild, moderate, or severe if the reduction in FEV_1_ (from the pre-exercise level) is > 10% but < 25%, > 25% but < 50%, or > 50%, respectively (Parsons et al., 2013).

### International Physical Activity Questionnaire

To evaluate the level of physical activity of the participants, we applied the International Physical Activity Questionnaire-Short Form (IPAQ-SF) (Matsudo et al., 2012). The IPAQ-SF comprises eight open questions designed to estimate the time spent per week in physical activity of different intensities (walking, moderate exercise, and vigorous exercise) and in sedentary activities (sitting). To classify the participants, we determined the relationship between the duration (minutes/day) and frequency (days/week) of physical activity reported on the IPAQ-SF.

Current guidelines for physical activity in childhood and adolescence recommend that young people engage in ≥ 60 minutes/day of moderate exercise on five or more days a week, for a weekly total of at least 300 minutes of moderate exercise or 150 minutes of vigorous exercise (World Health Organization, 2011). On the basis of that recommendation, *vis-à-vis* the responses on the IPAQ-SF, the participants were classified as active or inactive.

### CPET

The CPET was performed on a cycle ergometer coupled to a metabolic cart (MedGraphics Ultima CPX; Medical Graphics, Saint Paul, MN, United States). For the first 2 minutes, the participant was allowed to cycle with no resistance (at 0 w). Subsequently, the workload was increased once every minute and the participant was instructed to maintain a constant cycling speed of 50–70 rpm (Weisman et al., 2003). The progressive increase in workload was achieved with a linear ramp protocol, and the duration of the exercise ranged from 8 to 12 minutes, depending on the level of daily physical activity of the participant. Over the course of the test, the workload was increased by 5–15 w (Weisman et al., 2003).

The following variables were analyzed: workload (in watts) reached at peak exercise minute ventilation (VE), oxygen uptake (VO_2_), respiratory exchange ratio, carbon dioxide output (VCO_2_), the ventilatory equivalents (VE/VO_2_ and VE/VCO_2_) and the equivalent of the peak VO_2_ over the workload in watts (VO_2__peak_/w), the ratio between VO_2__peak_ and heart rate (VO_2__peak_/HR), and the ratio between VE and maximal voluntary ventilation (McNicholl et al., 2011). Those variables were measured from breath to breath, with analysis at every 20-second interval. The VO_2__peak_ was determined by calculating the average of the last 30 seconds of exercise, which was interrupted by signs of maximum exercise tolerance (Weisman et al., 2003). The predicted VO_2__peak_ was calculated with the equations proposed for children by Cooper et al. (1984):

for boys—*VO_2__*peak*_* (mL/min) = 52.8 × body weight − 303.4
for girls—*VO_2__*peak*_* (mL/min) = 28.5 × body weight + 288.2

Blood pressure, HR, and peripheral oxygen saturation were measured continuously, being recorded before the test, every 2 minutes during the test, immediately after the test, and 2 minutes after the test. Before and after the test, dyspnea and lower-limb fatigue were assessed with the modified Borg scale (Kovelis et al., 2008).

Participants were instructed to eat lightly on the day of the test, as well as not to drink coffee, tea, of caffeinated soda in the last 2 hours prior to the test. The asthma patients were instructed to suspend their use of long-acting bronchodilators for 12 hours prior to the test, of anticholinergics for 8 hours prior, of short-acting antihistamines for 24 hours prior, and of long-acting antihistamines for 72 hours prior (Parsons et al., 2013).

The CPET was considered maximal when the HR was ≥85% of the predicted value and the respiratory exchange ratio was appropriate (≥1.0 for children and ≥1.1 for adolescents), assuming that the patient had achieved the maximum effort (as evidenced by sweating and fatigue) during the test (Weisman et al., 2003). When the VE/maximal voluntary ventilation ratio was ≥80% and the VO_2__peak_ was <80% of the predicted value, the patient was categorized as having ventilatory limitation.

Patients showing an early plateau in the VO_2__peak_/HR ratio, not achieving 80% of the maximum predicted HR, not showing a ≥ 10 mmHg increase in systolic blood pressure at peak exercise, and showing a reduction in VO_2__peak_ to below 80% of the predicted value were categorized as having cardiac impairment. Hyperventilation was defined as a VO_2__peak_ ≥ 80% of the predicted value, an increased VE/VCO_2_ at the anaerobic threshold (>34), an elevated respiratory rate (>55 breaths/min), and normal peripheral oxygen saturation (>95%). Physical deconditioning was defined as a reduction in VO_2_ to below 80% of the predicted value, without evidence of cardiac impairment or ventilatory limitation (Weisman et al., 2003).

### Statistical Analysis

The variables were analyzed with the IBM SPSS Statistics software package, version 22.0 (IBM Corporation, Armonk, NY, United States). The distribution of the variables was evaluated with the Shapiro–Wilk test. Continuous variables are expressed as mean and standard deviation or as median and interquartile range, whereas categorical variables are expressed as absolute and relative frequencies. For comparisons between groups (patients vs. controls), the unpaired Student’s *t*-test or the Mann–Whitney test was used, as indicated. Values of *P* < 0.05 were considered significant.

## Results

Of the 60 patients in the cohort, 40 were excluded: 36 because they exhibited signs of a lack of asthma control other than exercise limitation (nighttime or daytime symptoms) or had used a short-acting bronchodilator in the last 4 weeks and 4 because they did not complete the protocol (because of difficulties in performing the CPET, in three, and because of exacerbation in the week of the exam, in one). Of the 21 controls recruited, two were excluded: one because of an abnormal electrocardiogram finding and one for not having met the criteria for a maximal CPET. Therefore, the final sample comprised 20 patients and 19 controls.

The clinical and functional characteristics of the study sample are shown in Table 1. Height and spirometric parameters were lower in the patients than in the controls. On the basis of the responses on the IPAQ-SF, all of the patients were classified as inactive, whereas most of the controls were classified as active. The allergic phenotype was predominant. Of the patients evaluated, 100% had allergic rhinitis and a positive skin test, as well as using high doses of inhaled corticosteroids, together with other controller medications, all of which are characteristic of severe refractory asthma. None of the patients were obese or were under continuous treatment with an oral corticosteroid.

**TABLE 1:** Characteristics of the population studied.

**Characteristic**	**Group**		***P***
	**Control**	**Asthma**	
	**(*n* = 19)**	**(*n* = 20)**	
Age (years), mean ± SD	13.2 ± 2.2	12.7 ± 2.9	0.53
Female, n (%)	14 (73.7)	14 (70.0)	0.99
Weight (kg), mean ± SD	49.9 ± 10.8	45.2 ± 13.9	0.25
Height (cm), mean ± SD	157.2 ± 10.2	149.5 ± 11.5	0.03
BMI (kg/m^2^), mean ± SD	19.9 ± 2.6	20.3 ± 4.5	0.72
Follow-up period (years), mean ± SD	−	4.6 ± 0.5	−
Nutritional status, n (%)			
Normal weight	19 (100)	20 (100)	—
Obese	0	0	—
IPAQ-SF classification, n (%)			
Inactive	7 (37)	20 (100)	< 0.001
Active	12 (63)	0	—
ACT/C-ACT score, median (IQR)	—	22 (18–23)	—
Medications			
Budesonide or equivalent (μg), mean ± SD	—	926 ± 299	—
ICS + LABA, n (%)	—	8 (40)	—
ICS + LABA + leukotriene receptor antagonist, n (%)	—	10 (50)	—
ICS + LABA + omalizumab + tiotropium, n (%)	—	2 (10)	—
Allergic test, n (%)			
Positive	—	20 (100)	—
*Dermatophagoides pteronyssinus* + *D. farinae*	—	6 (30)	—
*D. farinae* + *Blomia tropicalis*	—	1 (5)	—
*B. tropicalis* + *D. pteronyssinus* + *D. farinae*	—	6 (30)	—
Cat dander + *D. pteronyssinus* + *D. farinae*	—	4 (20)	—
Dander (Cat + Dog) + *B. tropicalis* + *D. pteronyssinus* + *D. farinae*	—	2 (10)	—
*B. tropicalis* + *D. pteronyssinus* + *D. farinae* + *Blattella germanica*	—	1 (5)	—
Comorbidities, n (%)			
Allergic rhinitis	—	11 (55)	—
Rhinitis + other comorbidities*	—	9 (45)	—
Spirometry			
CVF (L), mean ± SD	3.2 ± 0.2	2.8 ± 0.7	0.2
FVC (%)	98.7	94.5	0.3
CVF (Z), mean ± SD	0.4 ± 0.2	0.1 ± 0.4	0.4
FEV_1_ (L), mean ± SD	2.7 ± 0.7	2.2 ± 0.6	0.02
FEV_1_ (%), mean ± SD	101.5 ± 16.5	87.1 ± 18.8	0.01
FEV_1_ (Z), mean ± SD	−0.3 ± 1.2	−0.7 ± 1.7	0.01
FEF_25__–__75__%_ (L), mean ± SD	3.7 ± 1.3	2.2 ± 1.1	0.001
FEF_25__–__75__%_ (%), mean ± SD	95.5 ± 11.5	75.5 ± 31.2	0.01
FEF_25__–__75__%_ (Z), mean ± SD	0.2 ± 1.3	−1.2 ± 1.6	0.004
FEV_1_/FVC ratio (%), mean ± SD	89 ± 0.1	81 ± 0.1	0.02
FEV_1_/FVC ratio (Z), mean ± SD	−0.3 ± 1.4	−0.6 ± 1.4	0.04

ACT: asthma control test; BMI: body mass index; C-ACT: childhood asthma control test; FEF_25__–__75__%_: forced expiratory flow between 25 and 75% of forced vital capacity; FEV_1_: forced expiratory volume in one second; FVC: forced vital capacity; ICS + LABA: inhaled corticosteroid + long-acting β_2_ agonist; IPAQ-SF: International Physical Activity Questionnaire-Short Form; IQR: interquartile range.

*Atopic dermatitis, gastroesophageal reflux disease, emotional disorders, and behavioral disorders.

**FIGURE 1:**
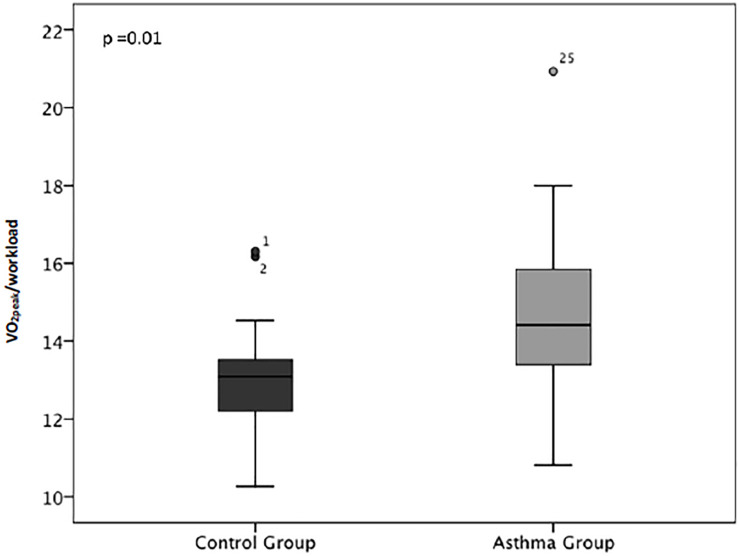
Comparison between the groups in terms of the ratio between peak oxygen uptake and the workload in watts (VO_2__peak_/workload) on cardiopulmonary exercise testing. Note that the patients with asthma consumed more oxygen, at the same or lower workload, than did the controls (*P* = 0.01).

The results of the CPET are shown in Table 2. All participants had maximal tests, and there were no complications during or after the tests. None of the patients showed stridor or changes in the shape of the spirometric curves. During the resting phase of the CPET, the variables were similar between the two groups. However, the workload (in watts) was lower, the test time therefore being shorter, in the patients than in the controls (*P* = 0.04). The VO_2__peak_/workload ratio was indicative of less ventilatory efficiency in the patients than in the controls (*P* = 0.01). Figure 1 shows the VO_2__peak_/workload ratio at peak exercise. The results of the CPET at anaerobic threshold (AT) are in a Supplementary Table S1.

**TABLE 2:** Results of cardiopulmonary exercise testing.

**Variable**	**Group**		***P***
	Control*	Asthma*	
	**(*n* = 19)**	**(*n* = 20)**	
**At rest**			
HR, bpm*	93.4 ± 12.6	88.5 ± 13.4	0.24
PAS, mmHg*	102.1 ± 9.7	99.2 ± 9.5	0.36
PAD, mmHg*	68.9 ± 8.0	65.0 ± 7.6	0.12
Borg dyspnea^†^	0 (0–0)	0 (0–0)	0.60
Borg LL fatigue^†^	0 (0–0)	0 (0–0)	0.79
**At peak exercise**			
Load reached, watts*	125.0 ± 26.6	104.0 ± 35.2	0.04
Test time, min*	10.4 ± 1.5	9.5 ± 1.4	0.06
HR, bpm*	182.9 ± 11.6	176.0 ± 15.9	0.13
VO_2__peak_/HR^†^	8 (8-10)	8.5 (7-9)	0.47
VO_2__peak_/workload*	13.0 ± 1.4	14.7 ± 2.3	0.01
SBP, mmHg*	143.1 ± 14.5	129.5 ± 11.9	0.03
DBP, mmHg*	72.1 ± 5.3	71.0 ± 5.5	0.53
VO_2_, mL/min/kg*	33.5 ± 7.7	32.6 ± 7.4	0.10
VO_2_,%*	84.4 ± 20.6	75.0 ± 14.3	0.26
RER^†^	1.20 (1.14–1.24)	1.15 (1.10–1.21)	0.04
VE, L/min*	64.5 ± 12.1	56.5 ± 13.4	0.06
VE/workload*	0.52 ± 0.07	0.56 ± 0.11	0.15
VE/MVV*	64.7 ± 16.3	68.5 ± 12.2	0.41
VE/VO_2_*	30.7 ± 3.1	32.4 ± 4.3	0.17
VE/VCO_2_*	34.2 ± 4.6	33.8 ± 3.30	0.27
Borg dyspnea^†^	7 (5–8)	5 (1–6)	0.07
Borg LL fatigue^†^	8 (5–9)	7 (1.5–9.1)	0.29

HR: heart rate; DBP: diastolic blood pressure; LL: lower limb; RER: respiratory exchange ratio; SBP: systolic blood pressure; VE: minute ventilation; VE/MVV: ratio between minute ventilation and maximal voluntary ventilation; VE/VO_2_: ventilatory equivalent for oxygen; VE/VCO_2_: ventilatory equivalent for carbon dioxide; VO_2__*peak*_: peak oxygen uptake.

*Mean ± SD.

*^†^Median (interquartile range).*

Among the participants in which a > 10% post-CPET reduction in FEV_1_ was observed, the mean reduction was 15.4 ± 6.9% in the asthma group and 15.2 ± 1.9% in the control group (Supplementary Table S2).

Table 3 shows the distributions of the participants in each of the categories established on the basis of the interpretation of the CPET. Among the 20 participants in the asthma group, we identified EIB in 6 (30%), deconditioning in 5 (25%), and EIB plus deconditioning in 6 (30%). The mean dose of inhaled corticosteroid was 1018 ± 373 μg/day among patients who presented physical deconditioning, compared with 800 ± 0 μg/day among those who did not (*P* = 0.082). The CPET identified the cause of the exercise limitation in 85% of the patients. Vocal cord dysfunction was not identified in any of the participants.

**TABLE 3:** Distributions of the participants in the categories established by the interpretation of the CPET.

**Interpretation of CPET**	**Group**	
	**Control**	**Asthma**
	**(*n* = 19)**	**(*n* = 20)**
EIB only, n (%)	3 (16)	6 (30)
Physical deconditioning only, n (%)	8 (42)	5 (25)
Physical deconditioning + EIB, n (%)	3 (16)	6 (30)
No abnormalities, n (%)	5 (26)	3 (15)
Ventilatory limitation, n (%)	0	0
Hyperventilation, n (%)	0	0
Cardiac impairment, n (%)	0	0

*CPET: cardiopulmonary exercise testing; EIB: exercise-induced bronchoconstriction.*

## Discussion

The most important finding of the present study was that the presence of physical deconditioning, with or without EIB, was the cause of the exercise limitation in 55% of the asthma patients in our sample. When we analyzed EIB in isolation, we found a prevalence of only 30%, lower than that reported in the literature, in which overall prevalence of EIB has been found to be 81% among individuals with severe asthma (de Aguiar et al., 2018) and approximately 60% among those with other forms of asthma, in population-based studies (Anthracopoulos et al., 2012) and observational cohort studies (Lin et al., 2019; van der Kamp et al., 2019). However, when we analyzed the combination of EIB and physical deconditioning, we found a prevalence of 55%, comparable to the prevalence of EIB reported in the literature.

Schiwe et al. (2019) and Villa et al. (2011) reported the prevalence of physical deconditioning, as assessed by calculating the VO_2_, to be 31 and 55%, respectively, among individuals with severe asthma, higher than the 25% observed in the present study for physical deconditioning in isolation. However, those authors did not evaluate EIB. As mechanisms for limiting exercise tolerance, physical deconditioning and EIB can act in isolation or in concert. In our study, we found that the frequency of EIB accompanied by physical deconditioning, as assessed through CPET, was twice as high in the patients as in the controls. In addition, of the 60% of our patients that presented EIB, half were physically deconditioned. As previously mentioned, none of the patients in our sample were obese.

In a population-based study conducted in Greece (Anthracopoulos et al., 2012), free running exercise challenge tests were employed in the evaluation of children 10-12 years of age. The authors found that the prevalence of EIB and the total energy expenditure were higher in the children who were moderately active or inactive than in those who were active, regardless of body mass index or asthma symptoms. Without implying causality, the authors suggested that a lack of physical activity predisposes to EIB. These results are in line with those of a study involving 26 children between 4 and 14 years of age with asthma in the Netherlands (van der Kamp et al., 2019), in which EIB was assessed with CPET and physical activity was assessed with accelerometers. Those authors found that the periods of vigorous activity were significantly shorter in children with EIB than in those without, emphasizing the need for a thorough clinical evaluation of exercise-induced symptoms when assessing physical activity in children with asthma. Lu et al. (2020) found that the chance of wheezing during physical exercise correlated positively with the time spent in sedentary behaviors and with physical deconditioning, as assessed by quantifying the time spent in continuous exercise. Other studies, also using objective methods, have shown that the level of physical activity does not differ between children with and without asthma (Cassim et al., 2016; Pike et al., 2019). In those studies, a lower level of physical activity was observed only in the children with asthma symptoms severe enough to warrant hospitalization. However, in another study (Sousa et al., 2014), the level of moderate physical activity was found to be comparable between children with and without asthma, even when those with severe asthma were included, although EIB was not evaluated in that study. In a prospective study of children in Sweden (Johansson et al., 2019), the prevalence of exercise-induced symptoms was found to double over a five-year period (from childhood into adolescence), and the level of physical activity was found to decrease over that same period.

The World Health Organization (2011) recommends that children engage in moderate to vigorous physical activity for a minimum of 60 minutes per day, although it does not provide any guidance regarding the exact proportion of vigorous activity (World Health Organization, 2011; van der Kamp et al., 2019), which has been shown to improve cardiorespiratory, muscular, and bone fitness (World Health Organization, 2011). That might explain our finding that 42% of the healthy controls were physically deconditioned, as determined with maximal CPET, despite the fact that our sample included children and adolescents who reported participating in recreational physical activities at least three times a week. It is likely that the children and adolescents evaluated were engaging in regular physical activity at an intensity lower than that required to generate fitness. Our results are consistent with data showing that 80% of healthy children and adolescents do not engage in physical activity at the levels recommended by the World Health Organization (2011). Data from the Brazilian National School-Based Adolescent Health Survey show that 65.6% of 9th grade students in Brazil engaged in fewer than 300 minutes of physical activity per week in 2015 (Brasil. Ministério da Saúde, 2015).

Children tend to alternate between short periods of vigorous physical activity and longer periods of light to moderate physical activity. Physiological indicators of physical conditioning are important biomarkers for monitoring overall cardiorespiratory, muscle, and bone health (World Health Organization, 2011). However, few studies have used CPET effectively to identify physical deconditioning (Vahlkvist and Pedersen, 2009; Villa et al., 2011; Schiwe et al., 2019).

In the present study, the patients with asthma consumed the same amount of oxygen but completed the test with a 25% lower workload in comparison with the controls, suggesting that individuals with asthma have less aerobic capacity. In addition, the patients reported less dyspnea, according to the Borg scale, than did the controls, possibly due to the lower workload they endured or the poor perception of symptoms that some asthma patients have (Schiwe et al., 2019).

Physical exercise is important to maintaining a high quality of life (Kansen et al., 2019). Dantas et al. (2014) evaluating the maternal perception of physical activity in adolescents with asthma, showed that 96% of mothers thought that physical activity was important. Of the mothers evaluated, 37% said that they imposed restrictions on their children’s physical activities (sports/games) even during periods when the children were not suffering from asthma exacerbations. In individuals with asthma, FEV_1_, before or after bronchodilator use, does not correlate with VO_2__*peak*_, the anaerobic threshold, or peripheral oxygen saturation (Kansen et al., 2019). The respiratory system, at least in individuals with mild asthma, is able to respond adequately to the demands for increased airflow during high-intensity aerobic exercise. That means that patients with asthma become less able and therefore less likely to become physically active and reach their maximum potential (Kansen et al., 2019).

In the present study, there were no significant differences between patients and controls regarding VO_2__peak_, in terms of the absolute value or percentage of the predicted value. The high proportion of volunteers with physical deconditioning in our control group may have been responsible for the lack of significance in the comparison between the two groups. Nevertheless, it is noteworthy that the patients ended the test with a lower workload for the same VO_2_, suggesting that physical deconditioning was less severe in the controls than in the patients. One surprising finding was that the patients with physical deconditioning alone used higher doses of inhaled corticosteroids than did those with EIB alone. That suggests that physicians are more likely to recognize EIB than physical deconditioning.

A finding of EIB can be indicative of a lack of asthma control, and adequate control of EIB has been associated with better fitness and with increased daily physical activity (Vahlkvist et al., 2010). Other studies have shown that children who have asthma that is well controlled, as assessed by the GINA criteria or ACT, regardless of the severity of asthma, have levels of physical activity similar to those of children without asthma (Sousa et al., 2014; Matsunaga et al., 2017). However, some authors question whether the ACT is able to detect reduced tolerance to moderate and vigorous activity, possibly only with a score below (Pike et al., 2019; van der Kamp et al., 2019).

In the present study, we used the ACT and the GINA criteria to assess asthma control, as previously described (Roxo et al., 2010; Oliveira et al., 2016; Page et al., 2017). The mean ACT score was 22, indicating good asthma control. However, the evaluation of the GINA criteria (by a pediatrician) revealed that the presence of exercise limitation, even in the absence of other symptoms, classified asthma as partially controlled. Our patients had not had an asthma attack in the last 12 months, and none needed to use a rescue bronchodilator after CPET or had oxygen desaturation. It is necessary to distinguish children and adolescents with recurrent asthma symptoms only during or after exercise, which are due to a lack of asthma control in general, from those with EIB or other conditions and from those with adequate control of other asthma symptoms. Only one study, involving adult patients, evaluated all possible mechanisms for exercise limitation (McNicholl et al., 2011). To our knowledge, ours is the first study to carry out such an evaluation in pediatric patients with severe asthma.

In the present study, we found no difference between the patients and the controls in relation to sex, body weight, or body mass index, and none of the individuals were classified as obese. However, the mean height was lower among the patients, although that difference had no impact on VO_2_, which varies depending on body weight and sex. However, the smaller stature of the patients with asthma in our sample might have been attributable to the high doses of inhaled corticosteroids that these patients had been using for many years. It is noteworthy that, even when using inhaled corticosteroids in combination with other controller medications, the patients had pulmonary function that was significantly impaired in comparison with that of the healthy controls. It is not known how physical deconditioning and physical inactivity in children with asthma will affect their lung function in adulthood (Lu et al., 2020).

Before stepping up the pharmacological treatment of a patient with asthma (McNicholl et al., 2011), every effort should be made to understand the reasons for exercise limitation. In addition to being a diagnostic aid, CPET provides objective parameters for physiotherapists to use in planning rehabilitation strategies (Weisman et al., 2003).

Physical activity has not been taken into consideration in the treatment of asthma (Panagiotou et al., 2020). However, the importance of physical activity for individuals with asthma is undeniable. The prescription of physical exercise for this population should take into account asthma attacks and exercise-induced asthma (Global Initiative for Asthma, 2020). It should be borne in mind that our patients were using several controller medications and nevertheless not achieving adequate control of asthma symptoms during exercise. Patients with severe asthma who have persistent complaints of symptoms during exercise, despite appropriate treatment, should be evaluated by objective methods to identify not only EIB but also physical deconditioning. Therefore, such evaluations should be conducted on an individual basis, given that physical deconditioning and other conditions can act in isolation or in concert with EIB as mechanisms for reducing exercise tolerance.

One limitation of this research was although the constant load CPET is a protocol described for EIB detection, the incremental load CPET protocol is also a valid option for such diagnosis (De Fuccio et al., 2005), added to the fact of determining other causes of exercise limitation other than EIB. Another limitation was the small sample size. However, that is to be expected because severe refractory asthma is a rare phenotype. Our patients were selected from a cohort of patients with severe asthma, with a well-established diagnosis and under regular follow-up for a long period, in which multiple methods were used in order to measure adherence, minimize exposure to allergens, and control comorbidities. From that cohort, we selected only those who complained of exercise limitation and who had no other symptoms indicative of a lack of asthma control. Given the cross-sectional study design, we cannot make inferences or draw conclusions regarding whether patients reduced their physical activity because they experienced EIB and thus became physically deconditioned or whether a sedentary lifestyle led to physical deconditioning that predisposed the patients to EIB. The power of our sample was calculated *a posteriori*, on the basis of the difference between asthma patients and controls in terms of the VO_2__peak_, with an effect size of 1.0 and the level of significance set at *P* = 0.05. With 19 controls and 20 patients, the power was 0.89.

## Conclusion

In conclusion, in patients with severe refractory asthma, persistent complaints of exercise limitation appear to be attributable not to ventilatory limitation but rather to physical deconditioning and EIB, the combination of the two being the most common profile. Therefore, exercise limitation does not appear to be indicative of a lack of asthma control.

## Data Availability Statement

The original contributions presented in the study are included in the article/Supplementary Material, further inquiries can be directed to the corresponding author/s.

## Ethics Statement

The studies involving human participants were reviewed and approved by Research Ethics Committee of the Federal University of Minas Gerais (Reference No. 3,720,392). Written informed consent to participate in this study was provided by the participants’ legal guardian/next of kin.

## Author Contributions

LL, EM, RF, and VG conceptualized the study. EM, LL, RF, and VG contributed to the study methodology. EM, LL, RF, VG, FL, MQ, and LO contributed to the formal analysis. LL, EM, and FL contributed to the software. All authors contributed to the data curation, validation, investigation, and writing (original draft preparation, and review and editing). All authors read and approved the final manuscript.

## Conflict of Interest

The authors declare that the research was conducted in the absence of any commercial or financial relationships that could be construed as a potential conflict of interest.
