# Supplementary material for: Exercise Limitation in Children and Adolescents With Severe Refractory Asthma: A Lack of Asthma Control?
Source: Front Physiol. 2021 Jan 26;11:620736. doi: 10.3389/fphys.2020.620736 (PMC7870485; doi:10.3389/fphys.2020.620736)
Supplement: Supplementary file 1 [file Table_1.pdf]

Table S1 Comparison of variables at the anaerobic threshold between groups

Variable	Group Control (n = 19)	Group Asthma (n = 20)	*P*
HR, bpm*	137.9 ± 12.4	135.7 ± 19.4	0.68
VO_2, mL/min/kg*	20.0 ± 6.1	18.9 ± 5.1	0.53
RER†	0.94 (0.89–0.96)	0.94 (0.91–0.99)	0.43
VE/VO_2*	29.1 ± 2.2	31.2 ± 4.0	0.06
VE/VCO_2*	31.5 ± 3.2	32.8 ± 3.6	0.26

HR: heart rate; RER: respiratory exchange ratio; VE/VO_2: ventilatory equivalent for oxygen; VE/VCO_2: ventilatory equivalent for carbon dioxide; VO_2: oxygen uptake.
*Mean ± SD.
†Median (interquartile range)
