# Supplementary material for: Exercise Limitation in Children and Adolescents With Severe Refractory Asthma: A Lack of Asthma Control?
Source: Front Physiol. 2021 Jan 26;11:620736. doi: 10.3389/fphys.2020.620736 (PMC7870485; doi:10.3389/fphys.2020.620736)
Supplement: Supplementary file 2 [file Table_2.pdf]

Table S2 Drop percentage of FEV_1 after CPET

	Group		
	Control (n = 6)	Asthma (n = 12)	p
FEV_1 after 5	-6.3 ± 4.5	-17.8 ± 9.5	0.014
FEV_1 after 10	-8.2 ± 5.8	-15.8 ± 12.2	0.173
FEV_1 after 15	-8.5 ± 5.5	-12.7 ± 10.8	0.381
FEV_1 after 20	-8.5 ± 5.7	-10.2 ± 9.9	0.699
FEV_1 after 30	-10.1 ± 4.3	-6.5 ± 6.0	0.213

FEV_1: forced expiratory volume in one second
